# S104. THE RELIABILITY AND VALIDITY OF THE CORE SCHIZOPHRENIA SYMPTOMS SCALE OF THE STANDARD FOR CLINICIANS’ INTERVIEW IN PSYCHIATRY

**DOI:** 10.1093/schbul/sby018.891

**Published:** 2018-04-01

**Authors:** Ahmed Aboraya

**Affiliations:** William R. Sharpe Jr. Hospital

## Abstract

**Background:**

Core schizophrenia symptoms (CSS) include delusions, hallucinations and disorganization and have been included in the diagnostic criteria of schizophrenia since Kraeplin (Kendler 2016). More than 40 subtypes of delusions, hallucinations and disorganization have been described as symptoms and signs of schizophrenia. There is a need to derive a short list of the core symptoms and signs of schizophrenia that are reliable, valid and useful for clinicians in clinical settings and clinical research.

**Methods:**

The Standard for Clinicians’ Interview in Psychiatry (SCIP) is a new diagnostic interview designed to be used by clinicians (psychiatrists and experienced mental health professionals) in clinical settings and clinical research. The SCIP is a valid and reliable tool and was tested in an international multisite study in three countries (USA, Canada and Egypt) between 2000 and 2012 (Aboraya, El-Missiry et al. 2014, Aboraya 2015, Aboraya 2016, Aboraya, Nasrallah et al. 2016). A total of 700 patients were interviewed at William R. Sharpe Jr. Hospital in Weston, West Virginia (670 patients) and Chestnut Ridge Center in Morgantown, West Virginia (30 patients). Mean patient age was 34, 59% male, 95% White and 34% had less than 12 years of education. The SCIP includes 38 items covering subtypes of delusions, hallucinations and disorganization. The 38 items were shortened by removing items with low prevalence, low sensitivity or low item-rest correlation (< 0.4). The reliability and validity of the remaining items was recalculated with repetitive iterations. The final model was developed with input from experts. The result is the Core Schizophrenia Symptoms (CSS) Scale which has 18 items: 6 items measuring hallucinations, 8 items measuring delusions and 4 items measuring disorganization. The items were scored with binary and Likert-type scales ranging from 0 to 3. The reliability of the CSS scale was measured using the kappa coefficient for inter-rater reliability of the CSS individual items and Cronbach’s alpha for internal consistency of the CSS dimension. The validity of the CSS scale was assessed using Receiver Operating Characteristic (ROC) curves to determine the best clinical cut-off point for the CSS scale that maximizes sensitivity and specificity of the scale against the SCIP diagnosis of schizophrenia (the reference standard).

**Results:**

Table (1) shows stable kappa values and standard error of 15 CSS items. Nine items have good reliability (kappa > 0.7), three items have fair reliability (kappa values range from 0.5 to 0.7) and three items have poor reliability (kappa < 0.5). Table (2) shows the internal consistency of the CSS dimension using Cronbach’s alpha and one-sided 95% confidence interval. The Cronbach’s alpha is 0.8317, indicating excellent internal consistency. Table (3) shows the sensitivity and specificity of the Core Schizophrenia Symptoms (CSS) scale. At a cut-off of one or more positive items, sensitivity is 95.06% and specificity is 88.94%; at a cut-off of two or more positive items, sensitivity is 90.12% and specificity is 89.39%.

**Discussion:**

The Core Schizophrenia Symptoms (CSS) Scale is reliable at the level of individual items and at the dimensional level. In addition, the CSS scale is a valid scale that differentiates between schizophrenia and non-schizophrenia cases in a clinical population.

